# Investigating Alfvénic wave propagation in coronal open-field regions

**DOI:** 10.1038/ncomms8813

**Published:** 2015-07-27

**Authors:** R. J. Morton, S. Tomczyk, R. Pinto

**Affiliations:** 1 Department of Mathematics and Information Sciences, Northumbria University, Newcastle Upon Tyne NE1 8ST, UK.; 2 High Altitude Observatory, National Center for Atmospheric Research, Boulder, Colorado 80307-3000, USA.; 3 UPS-OMP, IRAP, Université de Toulouse, 14 Avenue Edouard Belin -314000 Toulouse, France.; 4 CNRS, IRAP, 9 Avenue colonel Roche, BP 44346, F-31028 Toulouse, France.

## Abstract

The physical mechanisms behind accelerating solar and stellar winds are a long-standing astrophysical mystery, although recent breakthroughs have come from models invoking the turbulent dissipation of Alfvén waves. The existence of Alfvén waves far from the Sun has been known since the 1970s, and recently the presence of ubiquitous Alfvénic waves throughout the solar atmosphere has been confirmed. However, the presence of atmospheric Alfvénic waves does not, alone, provide sufficient support for wave-based models; the existence of counter-propagating Alfvénic waves is crucial for the development of turbulence. Here, we demonstrate that counter-propagating Alfvénic waves exist in open coronal magnetic fields and reveal key observational insights into the details of their generation, reflection in the upper atmosphere and outward propagation into the solar wind. The results enhance our knowledge of Alfvénic wave propagation in the solar atmosphere, providing support and constraints for some of the recent Alfvén wave turbulence models.

## Abstract (web summary)

Alfvénic waves are oscillations that occur in a plasma threaded by a magnetic field and their propagation, reflection and dissipation is believed to be partly responsible for the solar wind. Here, the authors observe the counter-propagating Alfvénic waves that most models require for solar-wind acceleration.

## Graphical abstract

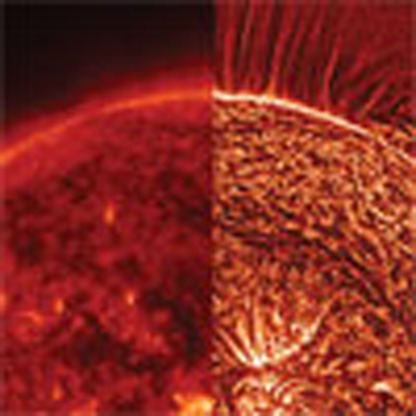

It is now well known that the fast solar wind originates from within open magnetic field regions in the Sun's atmosphere, for example, coronal holes, with wind acceleration beginning in the transition region and low corona^1^,^2^,^3^,^4^,^5^. However, the mechanism(s) responsible for heating the coronal plasma and accelerating the solar wind within the open fields is still unclear. A host of different physical models exist to explain this phenomena^6^ and one broad category of models relies on the propagation, reflection and dissipation of Alfvén waves^7^,^8^,^9^,^10^,^11^. Alfvén waves are perfect for the transport of energy as they are incompressible, making it difficult for their energy to be dissipated without the presence of large gradients in the Alfvén speed. This property should allow them to propagate out into the extended solar atmosphere and deposit their energy and momentum beyond the critical point and the point of temperature maximum, an important feature needed to explain the acceleration of fast solar winds^9^,^12^,^13^. These Alfvén waves would likely be generated towards the foot points of magnetic flux tubes, which are rooted in the photosphere and extend out into interplanetary space. In theory, after traversing the lower solar atmosphere, the waves reach the corona where they propagate and are partially reflected by the gradual variation in Alfvén speed, enabling the development of magnetohydrodynamic (MHD) wave turbulence^7^,^8^,^9^,^10^,^11^,^14^ and the subsequent dissipation of the energy transported by the waves.

The textbook Alfvén wave, first described by Hannes Alfvén, requires the existence of an infinite, homogenous plasma. In this scenario, other perturbations of the plasma can also be categorized neatly as slow and fast MHD waves. The distinct characteristics of Alfvén waves are that they are the sole propagator of vorticity perturbations and magnetic tension is the dominant restoring force, in addition to incompressibility. Conversely, slow and fast MHD waves are characterized by compressibility and do not perturb vorticity^15^. However, these characteristics are textbook ideas of MHD waves. In highly inhomogeneous plasmas, such as the solar atmosphere, MHD wave modes have mixed properties and cannot be so precisely categorized. The fine-scale structure in the solar atmosphere is frequently modelled as a collection of over-dense, magnetic waveguides. In such a setting, the wave energy is confined predominantly to the regions of enhanced density. Two wave modes in the system share characteristics reminiscent of the textbook Alfvén wave, namely the fundamental modes of the torsional Alfvén wave and the fast kink wave^15^. The torsional mode causes an incompressible rotation of magnetic surfaces. The fast kink wave generally has mixed properties. It is identified by a non-axisymmetric displacement of the waveguide (or a swaying motion) where magnetic tension is the dominant restoring force. The fast kink wave is also highly incompressible, especially in the limit where the wavelength is much larger than the transverse scale of the density inhomogeneity that defines the waveguide^16^. The kink mode also transports vorticity and shares a remarkable similarity with the surface Alfvén wave^15^. From theses considerations, we follow Goossens et al. (ref. 15, 16) and use the adjective Alfvénic to describe wave modes with characteristics similar to the textbook Alfvén waves. At present, it is unknown whether the models of Alfvén wave turbulence remain valid if the Alfvénic waves of inhomogeneous plasmas were used instead. The common characteristics shared by the waves could suggest that central features of the wave turbulence models may survive.

The existence of Alfvénic fluctuations far into the solar wind (at 1  AU) has been known for a number of years^17^ and their presence in the corona have been anticipated from the measurements of non-thermal line widths^18^,^19^,^20^,^21^,^22^,^23^,^24^,^25^. Recently, imaging and spectroscopic observations confirmed the presence of Alfvénic waves throughout the solar atmosphere^26^,^27^,^28^,^29^,^30^,^31^, with fast kink modes appearing omnipresent along fine-scale magnetic structures. The exact relationship between the motions encapsulated by the non-thermal width measurements and the wave motions seen in images is still unclear, typified by the apparent disparate measured amplitudes (and, hence, energetics). This is likely someway due to the complex dependence of non-thermal line widths on the resolution of the instrument, line of sight integration and the addition of different sources of motion, that is, flows and the multitude of MHD wave modes^32^,^33^,^34^.

Nevertheless, the associated flux of energy estimated to be carried by the Alfvénic waves at the coronal base appears to be sufficient to meet the requirements for heating the coronal plasma and/or solar wind acceleration. Although, again, there are still some remaining issues that need to be clarified with respect to the energy flux^31^,^35^,^36^,^37^ (also, see the Results section).

The presence of Alfvénic waves in the chromosphere and corona does not, however, provide sufficient support for acceleration of the solar wind via Alfvénic waves. For Alfvénic waves to be a viable option for wind acceleration, the presence of counter-propagating Alfvénic waves, over an extended frequency range, is crucial for MHD turbulence to occur in the open-field regions. Counter-propagation allows for the nonlinear interaction of the waves, which leads to a turbulent cascade. To date, only measurements of wave amplitudes and periods exist in such regions^18^,^19^,^20^,^21^,^22^,^23^,^24^,^25^,^30^,^31^. Further, current coronal observations of Alfvénic waves place only relatively simple constraints on the models, that is, non-thermal widths have provided frequency-integrated velocity amplitudes. More rigorous constraints can be placed on the models if insights are obtained into the evolution of the Alfvénic waves as they propagate from the corona out into the solar wind, along with measurements of key plasma parameters, for example, density, magnetic field strength.

In the following, these issues are explored through the analysis of both spectroscopic and imaging data from ground- and space-based observatories. We investigate whether the conditions in an open magnetic field region are suitable for MHD turbulence to develop, search for indications of the waves origin and whether the observed coronal Alfvénic waves are connected to those found far out in the solar wind. It is envisioned that the results will help constrain the variety of Alfvénic wave models. This is supported by an exploitation of the observed waves to probe some of the key plasma parameters of the open magnetic field region.

## Results

### The coronal multi-channel polarimeter

The Coronal Multi-Channel Polarimeter (CoMP)^38^ is used to analyse Doppler velocity fluctuations and their propagation in an open-field region located at the northern pole of the Sun. CoMP was the first instrument to confirm the ubiquity of Alfvénic waves in the corona^29^ and, still, has an unequalled ability to observe coronal velocity fluctuations, over a large field of view at high temporal cadence.

**Figure 1:**
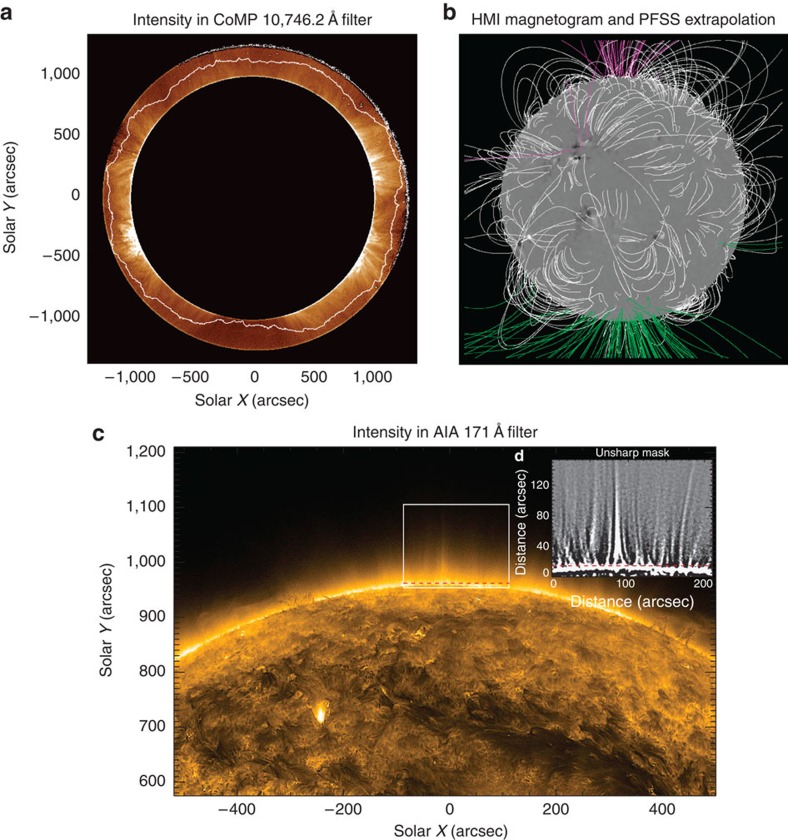
The solar corona. (**a**) An intensity image of the corona as seen in the infrared Fe XIII emission line, where the disk of the Sun has been occulted. The image is enhanced with a radial gradient filter for visual purposes only. The white contour in the corona demarcates the region for which Doppler velocities could be measured. (**b**) A magnetogram, based on Helioseismic and Magnetic Imager (HMI) data and evolved with the surface-flux assimilation model, demonstrates the photospheric magnetic field and is over-plotted with magnetic field lines from the PFSS potential field extrapolation. The purple and green lines show open-field regions of negative and positive polarity, while the white lines show closed field regions. (**c**) An enlarged image of the polar region as seen with SDO/AIA in the Fe IX coronal emission line. Fine-scale structures exist at the limb and are visible as elongated intensity enhancements. (**d**) An unsharp masked cut-out of a portion of the open-field region (box in **c**) clearly reveals this fine-scale magnetic structure extending radially away from the surface. The red dashed lines in **c** and **d** shows the position of the slit used to generate the time–distance diagram in Fig. 5a.

The Sun, on 27 March 2012, had two dominant open-field regions with the southern one more readily identifiable. The southern region is marked by the presence of a coronal hole that has extremely low emission in Fe XIII images of the corona obtained with CoMP (Fig. 1). This lack of emission means that the data preparation technique (see Methods) fails low in the coronal hole and as such there is no line centre intensity measurements (or Doppler velocities/widths). The region for which velocity measurements could be made is highlighted by the contour on the CoMP image in Fig. 1a. The northern open-field region is less obvious but there is reduced emission at the pole compared with the rest of the corona. The lack of an obvious coronal hole is also apparent in coronal Fe IX and XII emission from higher resolution, full disk images provided by the Solar Dynamics Observatory (SDO) Atmospheric Imaging Assembly (AIA), although radially orientated, field-aligned fine-scale magnetic structures are visible (Fig. 1c,d). The relationship of this fine-scale magnetic structure to plumes and inter-plumes is unclear from the current data set. We are able to say that they are much narrower than the classic plumes, which are reported to span ∼30 Mm (40 arcseconds) on average.

To confirm the presence of open fields in this region, we use the evolving surface-flux assimilation model and potential field source surface (PFSS) extrapolation tool^39^ (the extrapolation is shown in Fig. 1b). The southern coronal hole is seen in the extrapolation as a large open-field region that has some presence on the solar disk, while the northern open-field region is restricted to high latitudes. There is a direct correspondence between the locations of weak emission at low coronal heights in the CoMP data and the open-field lines in the PFSS extrapolation. We note that the visible structure in the region of interest remains practically unchanged throughout the observations, indicating little or no large-scale changes in the magnetic topology of the region.

### The existence of counter-propagating Alfvénic waves

**Figure 2:**
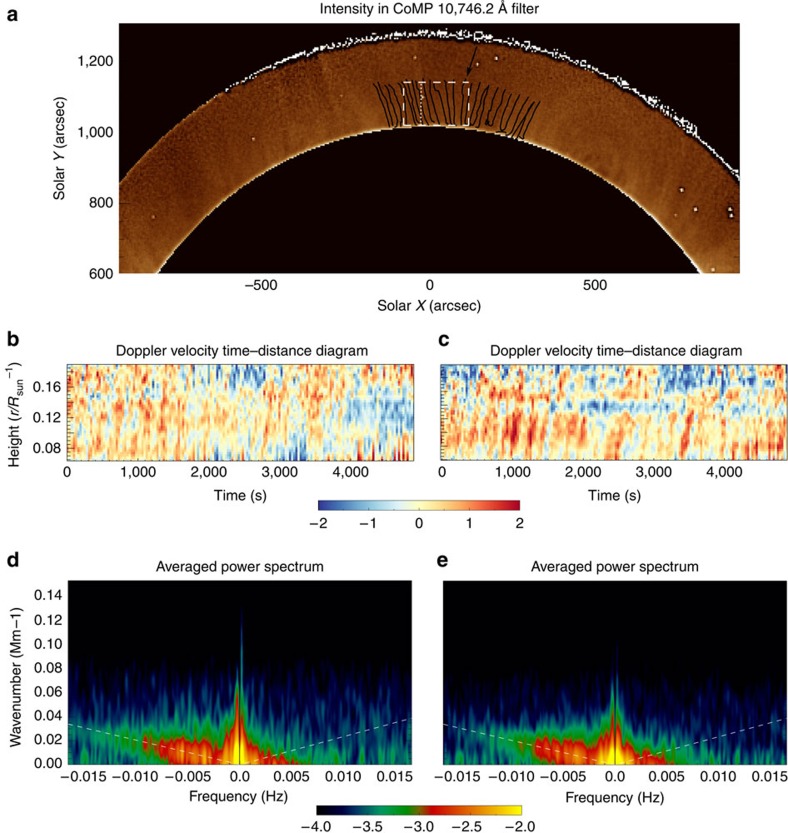
Propagating Alfvénic waves in the open-field region. (**a**) A close-up CoMP intensity image of the northern polar region that demonstrates the locations of the box (dashed white lines) and the tracks (black lines) used in the two methods for isolating propagating waves in the open-field region. The predominantly upwardly propagating waves can be seen in the Doppler velocity time–distance diagrams from the box (**b**) and from the track (**c**) methods, where the scale bar indicates the velocity (km s^−1^). The locations of these Doppler velocity time–distance diagrams are highlighted in **a** by the white dotted line (**b**) and black arrow (**c**). The time–distance diagrams are used to derive the corresponding frequency-wavenumber power plots for the box (**d**) and track (**e**) methods. A pronounced ridge of power in the negative frequency domain is clearly visible corresponding to the dominance of outward propagating waves. Crucially, the power plots also reveal the existence of inwardly propagating waves, but with reduced power. The scale bar indicates the power (km^2^ s^−2^) to the log base 10. The over-plotted white dashed lines show the calculated average inward and outward wave propagation velocities from the Doppler velocity time–distance diagrams.

We focus on the northern open-field region and examine the Doppler velocities in this part of the corona. It is evident from the data that there is a predominantly outward propagating Doppler signal in this region. To analyse the fluctuations in the Doppler velocities, we use two different methods to extract the data in the form of velocity time–distance diagrams (Fig. 2). The two independent methods are used to validate the analysis techniques and results of each other. For the first method, the crude assumption is made that the direction of potential wave propagation is strictly in the North–South direction. Individual Doppler velocity time–distance diagrams are generated from vertical strips of pixels in the boxed region (shown in Fig. 2a). The presence of the outward propagating Doppler velocity signals is visible in these diagrams and so is the periodic nature of these features, implying that the observed signals correspond to MHD waves.

**Figure 3:**
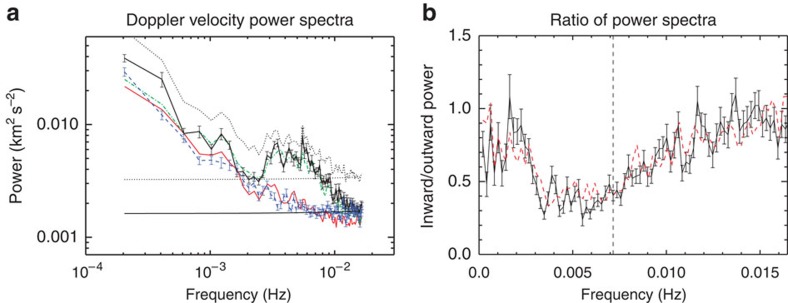
Average frequency power spectra and ratios of counter-propagating waves. The predominance of power in outward propagating waves (box method—black solid and track method—green dash-dot) is clear in the wavenumber-integrated power spectra (**a**). The spectra also provide insight into the power of the inward propagating waves (box method—blue dash and track method—red dash-triple dot). The total power spectrum is also shown for reference (dots). The horizontal dotted line is the estimate for the data noise associated with the total power spectra, while the solid horizontal line is the data noise split between inward and outward components. (**b**) The ratio of inward to outward power is displayed as a function of frequency and reveals a frequency-dependent trend suggestive of partial reflection of the waves in the corona. The vertical dashed line highlights the frequency after which noise begins to dominate the inward power spectra. The error bars in both plots show the s.e.m.

The power spectrum for the Doppler velocity time–distance diagrams is determined for a range of frequencies (*f*) and wavenumbers (*k*) in the vertical direction, and the outward and inward propagating signals are decomposed. To analyse the waves, both an *f–k* diagram (Fig. 2) and wavenumber-integrated power spectra are calculated (Fig. 3). A prominent ridge in the left half of the *f–k* diagram corresponding to negative frequencies confirms the dominance of outward propagating waves. No clear ridge exists for the inward propagating waves although there is significant power in the low frequency part of the spectrum. Such ridges have also been found in *f–k* diagrams for large quiescent coronal loops^40^.

The second method is a more refined version of the first, providing the *f–k* diagram, power spectra and, in addition, the propagation velocities of the waves (see Methods). The method explicitly determines the direction of propagation of the waves and assuming that these propagating waves are Alfvénic in nature, the direction of propagation defines the orientation of the magnetic field in the plane of the sky. Building up this picture of orientations over the entire atmosphere, the local direction of the magnetic field is determined.

Starting at a particular pixel, the direction of the magnetic field in the plane of sky is followed and tracks are built that are aligned with the direction of wave propagation. The tracks are almost vertical with respect to the solar disk, highlighting the radial structure of the magnetic field (black lines in Fig. 2). The Doppler velocity time series at each point along the track is then used to build Doppler velocity time–distance maps and, as with the preceding method, the *f–k* diagram (Fig. 2) and the *k*-integrated power spectra (Fig. 3) are obtained. The two methods show qualitatively similar results suggesting that the wave propagation direction is predominantly in the North–South direction, that is, radial for this small open-field region.

An estimate of the propagation speed of the waves is obtained from these Doppler velocity time–distance diagrams with a weighted average giving the typical speeds and standard deviation for outward propagation as 444±2 km s^−1^ and inward propagation as 365±76 km s^−1^. The propagation speed of Alfvénic waves is given by *c*_p_*≈ω/k* and this relationship is used to overplot the outward and inward velocities on the *f–k* diagrams in Fig. 2. Good agreement between the location of the ridge and the estimated outward propagation speed is clear, giving confidence in the values. These speeds are significantly greater than the sound speed for a 1.6 MK plasma *c*_s_∼150 km s^−1^ and, hence, the observed waves are likely Alfvénic.

As mentioned, counter-propagating waves are a crucial component of Alfvén wave heating/acceleration models if MHD wave turbulence is to develop^14^,^41^, generating the small scales necessary for the dissipation of Alfvénic wave energy through their interaction. The power spectra obtained here provide evidence for the presence of both outward and inward propagating Alfvénic waves in open-field regions, suggesting that the Alfvénic waves are at least partially reflected in the open magnetic field regions of corona, or further out.

The inwardly propagating waves can be seen to have significantly less power, both in the *f–k* diagrams (Fig. 2c,d) and the wavenumber-integrated power spectra (Fig. 3a). In Fig. 3b, the ratio of inward to outward wave power is given as a function of frequency. The ratio reveals a frequency dependence of the power ratio, potentially indicative of wave reflection in the corona. The results suggest (for *f*<6 mHz) that the low frequency waves are more readily reflected than high frequency waves, which is to be expected for coronal Alfvén waves^7^,^9^. However, the ratio begins to increase after *f*∼6–7 mHz. The increase in the ratio for these higher frequencies is likely related to the inward power being dominated by noise for *f*>6 mHz. The noise in the data has a flat power spectrum and the continued decrease in the outward wave power with frequency >6 mHz leads to the increase in the ratio. The ratio flattens off for *f*>10 mHz, a sign that the outward power spectra also becomes dominated by the noise. The influence of noise is confirmed upon estimating the contribution of the data noise to the power spectra.

### Connecting the coronal waves to the solar wind

The power spectra of the outward and inward propagating waves are found to have a slope of *∼1/f* (see Table 1 for exact values) and an enhancement of power at ∼3–5 mHz. The functional *1/f* form of the coronal power spectra is also found in magnetic and velocity fluctuation power spectra observed in solar wind streams in the heliosphere at 0.3 AU (64 
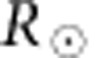
)^42^,^43^. The similarity between the slopes of the coronal and wind power spectra lends support to the ideas presented in ref. 43, which suggest that the shape of the wind spectrum is already set in the low solar atmosphere, at least in the low corona and transported outwards.

Interestingly, the presence of an enhancement in power is observed at ∼1–2 mHz in some solar wind spectra^42^, similar to the enhancement in the coronal power spectra at frequencies of 3–5 mHz. This may imply that the features seen in solar wind spectra are potential remnants of the functional form of the power spectra set in the low solar atmosphere (<1.3 
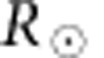
). The frequency shift of this feature between the two spectra, that is, coronal and at 0.3 AU, may be explained by the stretching of the perpendicular wavenumber scale with height owing to the radial expansion of the magnetic field combined with solar wind acceleration and magnetic field line rotation^44^.

### Evidence *p*-modes play a role in Alfvénic wave generation

The enhancement of power seen in the coronal power spectra is of interest in its own right. The frequency range occupied by the power enhancement is coincidental with the frequency range of peak power of *p*-modes and has also been observed in CoMP power spectra for large quiescent coronal loops^40^. This leads us to suspect that *p*-modes play an important role in the generation of Alfvénic waves in open-field regions, as well as in the quiescent Sun.

Theoretical considerations support the idea that *p*-modes can be guided into the corona by the magnetic field. In particular, mode conversion would play a dominant role in generating coronal Alfvénic waves^45^. At some height in the lower solar atmosphere, dependent upon the local pressure and magnetic field, there will exist a canopy where the gas pressure and magnetic pressure are approximately equal in magnitude (*β=8πp/B*^*2*^*≈1*). At the canopy, a transformation from slow to fast magnetoacoustic waves can occur, with the efficiency of this transformation dependent upon the angle between the magnetic field and the direction of wave propagation. This phenomenon has been well studied in a variety of magnetic field configurations and plasma conditions^46^,^47^,^48^,^49^. Although slow magnetoacoustic modes above this canopy are expected to steepen and shock owing to the rise in temperature in the upper chromosphere, the fast magnetoacoustic waves are reflected due to the steep gradient in Alfvén speed at the transition region. In the case when the gravity, magnetic field and wave vector of the fast magnetoacoustic wave are not co-planar, then there is a coupling of the fast wave to the Alfvén wave^45^,^50^,^51^,^52^. This coupling enables wave energy to cross the transition region and propagate into the corona as an Alfvén wave. Finally, regions of strong magnetic field provide the necessary conditions for *p*-modes to leak out from the interior into the atmosphere^53^,^54^,^55^, where they can then be converted via various methods of mode coupling. This is of particular importance as the open coronal fields are generally thought to be rooted in the kilo-Gauss faculae that form the magnetic network in the photosphere^56^,^57^. It has been estimated that a sufficient amount of energy can be converted from *p*-modes to coronal Alfvén waves to explain the approximate energy content of observed Alfvénic motions in the corona^45^.

We note that the discussed theoretical work^45^,^50^,^51^,^52^ has only been undertaken for a homogenous atmosphere, that is, the excitation of classical Alfvén waves. It is still unknown whether *p*-modes could excite Alfvénic waves in a corona with a cross-field density structuring. However, it is clear from the theory that vorticity perturbations must be excited in the corona by the mode conversion at the transition region; otherwise Alfvén waves would not be produced. In the case of a structured corona, any generated vorticity at the transition region must also be transported and the Alfvénic modes would seemingly be the best choice for this. The observed enhanced power of the coronal Alfvénic waves coincident with the frequency range of the dominant *p*-mode power may support this but further investigation is required for the confirmation of this hypothesis.

### Probing open-field regions with solar magneto-seismology

Moving away from the power spectra, another interesting aspect observed is that the propagation velocities of the outward waves is greater than the inward. Under the assumption that there exists a background outflow, in this case most likely the solar wind, then a simplified relationship describing the Alfvénic waves can be obtained (see Methods),


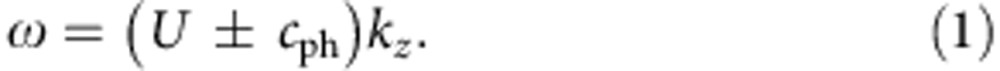


Here, *c*_ph_ is the phase speed of the wave, *U* is the flow speed and *z* is the direction parallel to the magnetic field. The propagation speed of the outward (plus) and inward (minus) waves is modified by the presence of the flow. Hence, outward and inward wave speed estimates obtained earlier from the Doppler velocity time–distance diagrams suggest the presence of an outward moving flow with an average speed *U*=31±7 km s^−1^. This value is comparable to the spectroscopic estimate of outflows in open-field regions obtained from the transition region and low coronal emission lines^58^.

**Figure 4:**
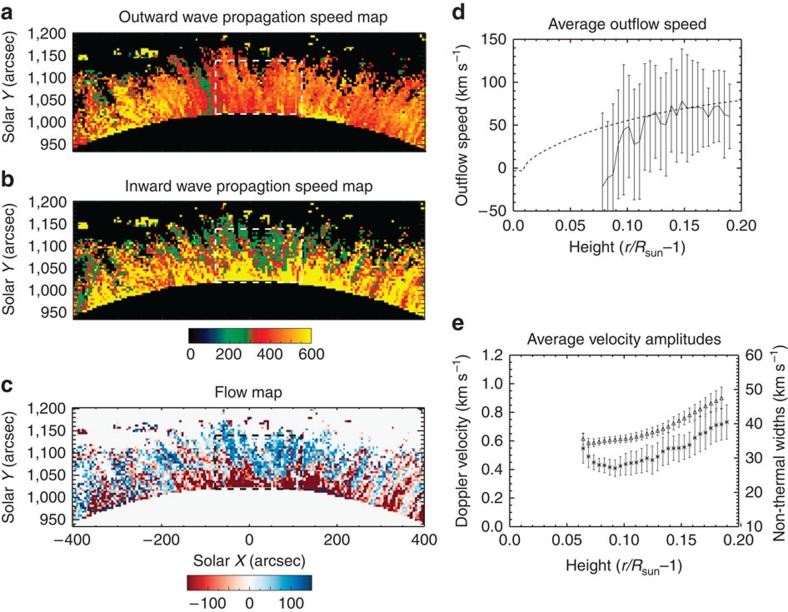
Measurements of the flow speed along the open fields. The measured propagation speed for the outward (**a**) and inward (**b**) Alfvénic waves can be used to determine the flow speed of plasma along the magnetic field (**c**). The black and white boxes in the propagation and flow maps mark the region that is used for determining the average values in the following panels. The scale bars indicate propagation speed (km s^−1^) and flow speed (km s^−1^). The open-field region is dominated by outflowing plasma (**d**), which is likely the beginnings of the solar wind and is comparable to outflows obtained from hydrodynamic wind models (dashed line). The error bars show the standard deviation of the average flow. (**e**) The RMS Doppler velocity (stars) and non-thermal widths (triangles) are shown as a function of height revealing that the Alfvénic wave amplitude increases as they propagate away from the limb. The error bars show the s.d. of the respective quantities.

This basic process is taken further. Following the same principles outlined for creating Doppler velocity time–distance diagrams, estimates for the outward and inward propagation velocities throughout the corona are obtained (Fig. 4a,b, see Methods for details). Subsequently, a flow map for the northern polar region is derived (Fig. 4c), which reveals a scene that is dominated by outflows, as should be expected if the open magnetic fields are a source region for the solar wind. The flow is seen to accelerate as a function of height (Fig. 4d), tending towards a value of 60–70 km s^−1^ at 1.2 
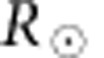
. The observed acceleration of the wind above ∼1.09 
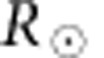
 is consistent with predictions of wind acceleration in the low corona found from solutions of one-dimensional hydrodynamic solar wind models (ref. 59—wind speed from the model is shown in Fig. 4d). Interestingly, the wind speed values from the model at ∼1.4 
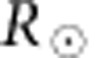
 (not shown in the figure) are comparable to previously measured outflow speeds in open-field regions at the corresponding height^4^. Note, the measured variation of average flow speed from inflow to outflow below 1.09 
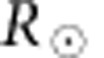
 is thought to be an artefact of erroneously high-estimated inward propagation speeds, hence inflow speeds, near the occulting disk (see Methods).

**Figure 5:**
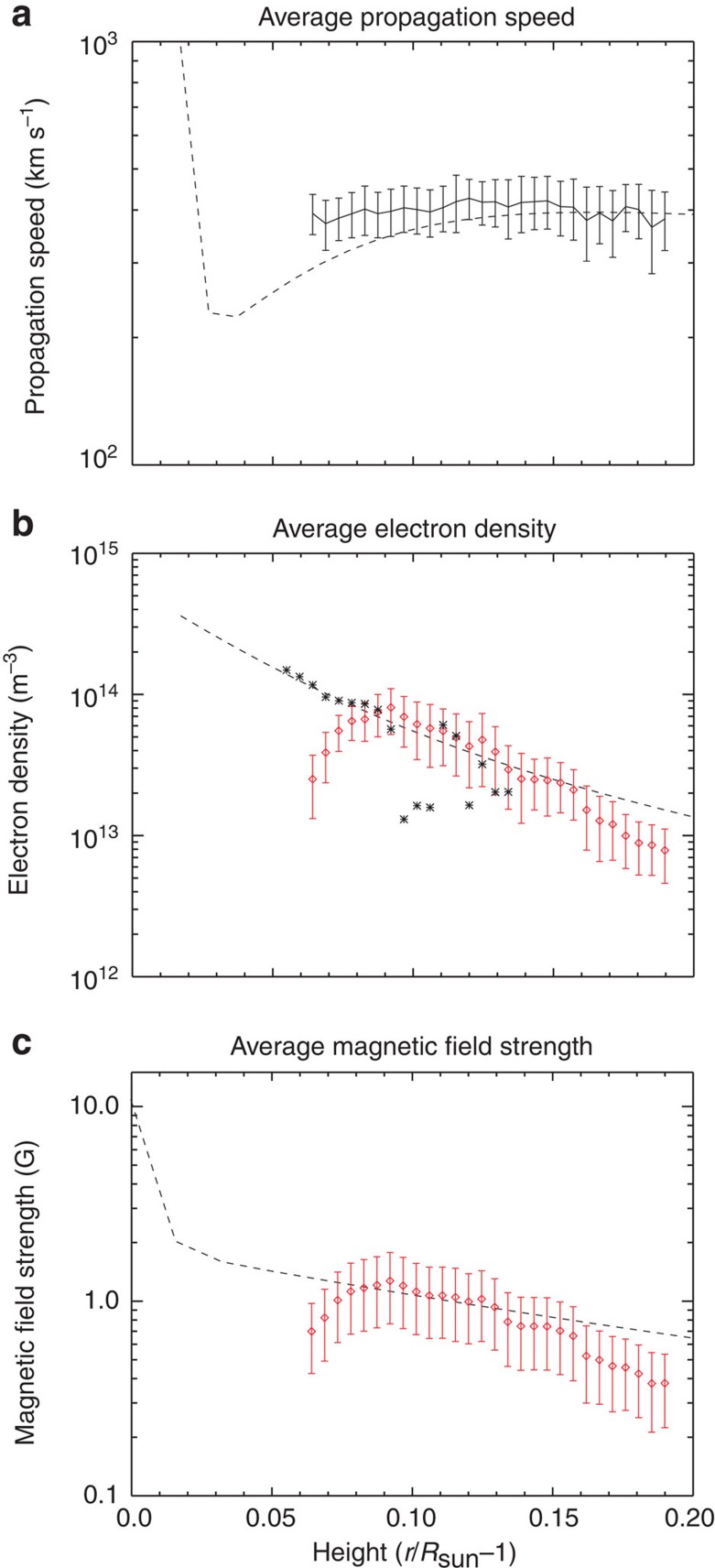
Physical properties of the open-field region. Multiple complementary techniques are used to estimate the physical properties of the magnetized plasma. (**a**) The measured outward propagation speed (solid line), averaged over the boxed region in Fig. 4a, is compared with the estimated propagation speed calculated from the analytic density profile (dashed line in **b**) and PFSS magnetic field (dashed line in **c**). (**b**) The electron density estimates from CoMP (stars) is shown and is compared with the density profiles obtained from magneto-seismology of solar atmosphere (red diamonds) and an analytic density profile (dashed line—ref. 7). (**c**) The magnetic field strength profile from the magneto-seismology (red diamonds) is compared with the magnetic field strength estimated from the PFSS potential field extrapolation (dashed line). The error bars in all plots are the s.d. of the respective quantities.

The measured properties of the Alfvénic waves, that is, amplitude and propagation speed, are further exploited to gain estimates for the gradients of magnetic field strength and density in the open-field region (see Methods). These gradients are shown in combination with electron density measurements available from CoMP, an analytic model of electron densities in an open magnetic field region from measurements in white light^7^ and the magnetic field estimated from the PFSS extrapolation model (Fig. 5a–c).

The estimated electron density from CoMP is found to follow the expected trend from the analytic model and this is supported by the density gradient obtained from the magneto-seismological inversion. In addition, the magnetic field gradients from the magneto-seismology show good agreement with that predicted by the PFSS. The quality of the agreement between the different measures for the plasma parameters suggests that the physical properties of this particular open magnetic field region are well constrained by this set of observations.

### Energetics of the waves

**Figure 6:**
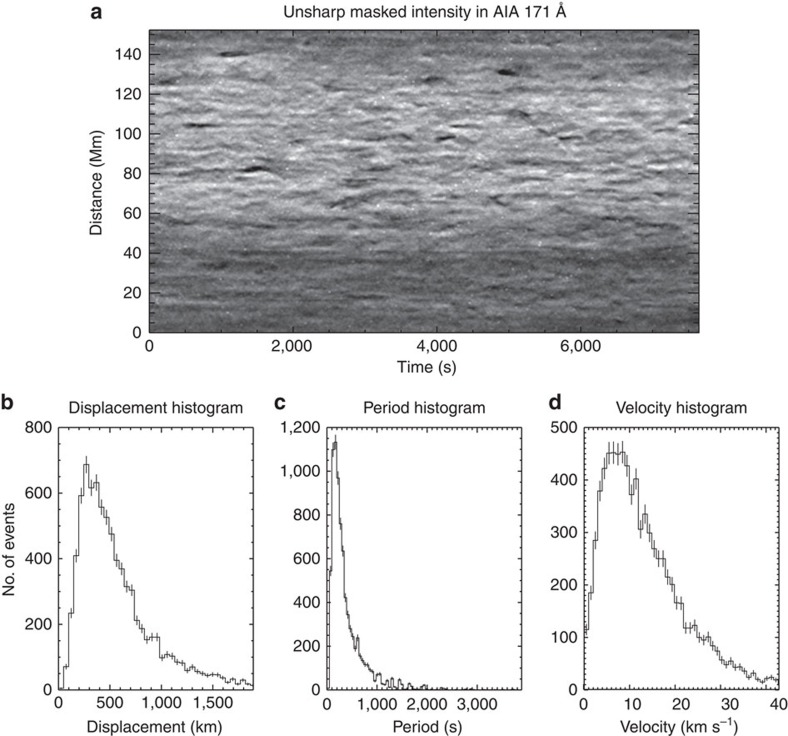
Direct observations of Alfvénic waves with SDO. An example time–distance diagram (location shown in Fig. 1c,d) reveals that the fine-scale magnetic structure in the open-field region sways periodically (**a**), and this motion is interpreted from MHD wave theory as the kink mode. These Alfvénic waves observed with SDO contribute to both the CoMP Doppler velocities and non-thermal widths. The wave motion is measured directly (**b**–**d**) and the mean values with s.e.m. are 590±5 km for displacement amplitude, 470±6 s for the period and 14.7±0.2 km s^−1^ for the velocity amplitude. Error bars show the sample s.d. for each bin in the histogram.

Now, combining CoMP and SDO observations, we are able to provide insight and constraints on the energetics of the observed Alfvénic waves. Using SDO, we measure the swaying motions of the fine-scale structures (Fig. 1d) at a height of ∼1.01 
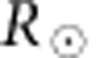
 (Fig. 6), which is interpreted in terms of the MHD kink wave mode^28^,^31^,^60^. It is likely to be these swaying motions that correspond to the periodic fluctuations of the CoMP Doppler velocities^33^. The observational signature of torsional motions is oppositely directed Doppler velocities on either side of the fine structure. Owing to large spatial resolution of CoMP and its inability to resolve the finest structure in the corona, these oppositely directed velocities will average to zero and have a negligible contribution to the measured Doppler velocities. The kink motions are observed to be periodic, with the distribution of periods peaking at ∼300 s (3.3 mHz)—coinciding with the frequency range of the enhanced power in the CoMP observations (Fig. 3a). The measured velocity amplitudes have an average value of 14 km s^−1^. This value is significantly larger than CoMP Doppler velocity amplitudes (<1 km s^−1^—Fig. 4e), although the poor spatial resolution of CoMP is known to lead to a significant underestimation of the Doppler velocities due to bulk plasma motions^33^,^34^. Conversely, the measurements from SDO data are almost a third of the value of the non-thermal velocities (Fig. 4e) that CoMP measures (and other instruments^20^,^21^,^23^,^24^,^25^)—although velocity amplitudes up to 40 km s^−1^ are found in the SDO measurements (Fig. 6). The reasons for the larger amplitudes inferred from the non-thermal widths are still unclear. Should unresolved torsional Alfvénic motions be present along fine structure, it would then be expected that they would contribute to non-thermal line broadening^61^. In addition, unresolved kink motions with small displacement amplitudes (so as to be also unobservable with SDO) would also contribute.

The upper limit on the energy flux of propagating Alfvénic waves^37^ is given by *F≈2<ρ>v*_rms_^*2*^*c*_ph_*δ*, we estimate the contribution of the kink MHD waves is on the order of ∼50*δ *W m^−2^, where *v*_rms_ is the root mean square velocity amplitude and *<ρ>*=9.5 × 10^−13^ kg m^−3^ is the average density at 1.01 
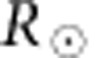
 extrapolated from CoMP electron density measurements. A phase speed of 250 km s^−1^ is also used^30^. The factor *δ* corresponds to the filling factor of the waveguides in the observed region. The value of *δ* is unclear although it likely <50% (see Fig. 1). The total wave energy including both kink and torsional motions may be best calculated by the non-thermal widths, giving a total value of ∼300*δ* W m^−2^ at 1.06 
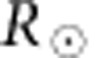
 *(<ρ>=*2.7 × 10^−13^ kg m^−3^, *c*_ph_=450 km s^−1^)—which can meet the energy flux requirements for accelerating the solar wind assuming *δ*∼50% (ref. 62). However, this number should be treated with some caution as it is uncertain exactly what physical phenomena are contributing to the non-thermal broadening^25^,^33^.

## Discussion

The initial study presented here demonstrates the potential for using Doppler velocity time series of the corona to understand Alfvénic wave propagation through the solar atmosphere and connecting the dynamics of the corona to the solar wind. The results reveal the existence of both outward and inward propagating Alfvénic waves in open magnetic field regions—a crucial element required if MHD wave turbulence is to develop. We remind the reader that it is presently unknown whether the details of the nonlinear interaction of fast kink waves are similar to those of Alfvén waves. Although the kink waves are Alfvénic, the turbulent cascade of vorticity to small scales through their nonlinear interaction seems entirely plausible. Alternatively, the kink waves are subject to mode conversion via resonant absorption^15^ and the energy could be transferred to torsional Alfvén waves before it is cascaded to higher frequencies.

Further, the ratio of outward to inward wave power demonstrates a frequency dependence that is suggestive of the waves being partially reflected in the corona, that is, a decreasing proportion of inward power to outward power with increasing frequency, which is also a feature in the models of Alfvénic wave propagation.

In addition to this, the power spectra reveal insights into the generation of the waves and their propagation. The slope of the power spectra for the Alfvénic waves shows a 1/*f* functional form that is comparable to previously measured solar wind spectra at 0.3 AU. This implies that the waves propagate outwards from the corona into the solar wind and at least partially maintain the shape of the power spectra^44^. Moreover, the observations reveal the presence of enhanced power centred on the frequencies routinely associated with peak *p*-mode power. Current theoretical models suggest that the acoustic waves generated in the solar interior can generate coronal Alfvénic waves through mode conversion^45^,^50^. We again highlight that these models use a homogenous corona, which raises questions about whether these results are directly applicable to the observations presented here. However, the current models suggest that vorticity is generated in the corona and the fast kink modes are one of the key transporters of vorticity in an inhomogeneous plasma^15^. This may advocate that *p*-modes could play a dominant role in generating coronal Alfvénic waves in the frequency range neighbouring 3 mHz. The role of acoustic waves is excluded from certain surface to heliosphere solar wind acceleration models dependent on Alfvén waves due to a simplifying assumption of incompressibility throughout the system (note that not all Alfvén wave models assume incompressibility). The indication that *p*-modes could play an important role in wave generation suggests that this assumption would neglect a fundamental physical process.

Currently, the estimates of energy flux from observable Alfvénic (that is, kink) waves in open-field regions advocate that there is insufficient energy to meet the demands for solar wind acceleration^31^. Although, CoMP measurements of the 10,747 Å line profile (and a host of other coronal emission line profile measurements from different instruments) reveal that the velocity amplitudes from non-thermal widths imply otherwise. This would indicate that only by including contributions from both resolved and unresolved (and both kink and torsional) Alfvénic waves can the energy flux requirement for solar wind acceleration be potentially met exclusively by waves. Further work is clearly needed to deconstruct the motions that comprise the non-thermal widths and provide a clear estimate for the energy flux of all Alfvénic waves.

This study also demonstrates the power of magneto-seismology of the solar atmosphere to probe the corona. Using the measured properties of the waves, evidence for the origins of the solar wind are seen in flow maps and significant constraints can be placed on other physical properties of the open magnetic field region, that is, magnetic field strength, density and the gradients of the two quantities. It is hoped that the measurements of the plasma parameters, along with the measured wave properties, for example, amplitude and power spectra, should provide a significant test scenario for models of solar wind acceleration, especially those which focus on MHD Alfvénic wave turbulence.

## Methods

### Observations and data reduction

The data used here were obtained with the CoMP^38^ instrument on 27 March 2012 at 18:51:02 UT to 20:13:02 UT. The details of the acquisition and reduction of the data are fully described in refs 29, 38, 40 and we make use of the final data product that has a cadence of 30 s and a pixel size of 4*″*.46 (3,234 km). The final data product is intensities at three infrared wavelengths (10,745.0 Å—*I*_1_, 10,746.2 Å—*I*_2_ and 10,747.4 Å—*I*_3_), which are positions centred on the Fe XIII emission line at 10,747 Å. The filter has a bandpass of 1.3 Å. Following ref. 63, for each pixel in the CoMP field of view in each time frame, we calculate the central intensity, Doppler shift and Doppler width of the line profile using an analytic fit of a Gaussian to the intensity values at each wavelength position.

We also make use of SDO AIA^64^ and the Helioseismic and Magnetic Imager^65^ data, which are taken from 18:00 UT to 20:10 UT and processed following standard procedures.

### Velocity time–distance maps

Here, we describe the two complementary methods for analysing the wave power spectra.

For the first method, a box is defined over the northern region, which is 200*″* wide and 125*″* tall (shown in Fig. 2). We make the crude assumption that, in the boxed region, the direction of potential wave propagation is strictly in the North–South direction. Displayed in Fig. 2 is an example of Doppler velocity time–distance diagram from a strip of pixels within the box, orientated in the vertical direction and one pixel wide. The presence of the outward propagating Doppler velocity signals is visible and so is the periodic nature of these features.

For each pixel in the box, the time-averaged value of the velocity is subtracted from the time series. Then, for each vertical strip in the box, the time–distance diagram is subjected to a two-dimensional Hamming window before a two-dimensional Fast Fourier Transform (FFT) is performed and the power for a range of frequencies and wavenumbers in the vertical direction is calculated. The outward propagating motions are decomposed from the inward propagating motions. Averaging the outward and inward power over the different strips, the power is integrated over wavenumber (*k*) and the outward and inward power spectra are determined (Fig. 3). The spectra are fit with a power law of the form *10*^*a*^ *f*^*b*^. The outward spectra are fit in two frequency ranges while, the inward wave power spectra are not fit for the high frequency ranges due to the power spectra being predominantly noise in this regime. The results for the fits are given in Table 1.

It is highly instructive to examine the averaged *f–k* diagram obtainable from the time–distance diagrams. To improve the frequency/wavenumber resolution in the *f–k* diagrams, the mean subtracted and windowed time–distance diagrams are padded with zeros before the FFT is performed. The results are shown in Fig. 2d.

The second method used is a more refined version of the first, providing the *f–k* power spectra and, in addition, the propagation velocities of the waves. This technique was demonstrated in ref. 40 and involves the tracking of wave packets via a coherence approach. In brief, for each pixel in the CoMP data, the time series is filtered at 3.5 mHz and the coherence of this filtered time series with the filtered time series in neighbouring pixels is calculated. This provides islands of high coherence, typically elongated in a particular direction that corresponds to the angle of wave propagation. Assuming that these propagating waves are Alfvénic, the direction of propagation defines the orientation of the magnetic field in the plane of the sky. It has been demonstrated that this technique provides magnetic field orientations that are in excellent agreement with the CoMP magnetic field measurements from the linear polarization of the 10,747 Å emission line^40^, and, hence, supporting the assumption of Alfvénic waves.

Starting at a particular pixel, we follow the direction of the magnetic field in the plane of sky using the calculated wave angles. Doing so allows tracks to be built that are aligned with the direction of wave propagation. We select 36 of these tracks that lie in the northern open-field region and these tracks are over-plotted on an intensity image in Fig. 2. The velocity time series at each point along the track is then extracted via cubic interpolation. An example of one of the time–distance diagrams from a track is shown in Fig. 2c. As with the first method, these Doppler velocity time–distance diagrams are used to create an average *f–k* diagram (Fig. 2e) and the *k*-integrated power spectra (Fig. 3a). Note that the power spectra and ratios in Fig. 3 are calculated from the original Doppler velocity time–distance diagrams, zero padding was not used to create these figures.

Note that the power in the *f–k* plots in Fig. 2 has been clipped between the given ranges. This leads to no loss of useful information as the signal is progressively noisier for larger *k* values and is done purely for aesthetic reasons.

### Propagation speed calculation

An estimate of the propagation speed of the waves is obtained from the Doppler velocity time–distance diagrams. The procedure is based on the technique described in ref. 40, so we give a brief overview of the technique and additions made to the process.

For each pixel, a time–distance diagram is created from tracks that are built using the wave propagation angles. Each time–distance diagram is subject to an FFT and then decomposed into inward and outward parts, both of which are then subject to an inverse FFT. The following is applied to both the inward and outward time series separately. The time series central pixel along the track is used as the ‘master time series' and is cross-correlated with the time series of the other pixels along the track to find values for the phase lag between the series. The lag values between the different time-series are fit with a linear function, which is then used to align the time series to the ‘master time series'. The aligned series are then averaged to create a higher signal-to-noise ‘master time series'. This new ‘master time series' is then cross-correlated with the time series of the other pixels along the track to get improved estimates on the lag values. A sub-resolution estimate of the phase lag is obtained by fitting the cross-correlation peak with a parabola. This process still leaves a number of high error or outlier lag data points that can negatively influence the result. To remove these points, two steps are applied. First, a null hypothesis test is performed. The value of the cross-correlation of any of the two series is tested to see whether the two series are uncorrelated and each series is not auto-correlated. For two such series with populations that are normally distributed, the sample cross-correlations would have zero mean and a variance of *1/N*, *N* being the number of samples. For a 99.7% confidence interval, this assumes that the null hypothesis is incorrect if the cross-correlation value is >*2.58/√N*. Lag data points with cross-correlation values below the cut-off are discarded. Having removed these data points, a linear fit to the lag data points is performed. From the residuals to the fit, the residual sample standard deviation is calculated. Here, the second cut is applied. Assuming the residuals are sampled from a normal distribution, a 99.7% significance level is calculated using *t*-distribution statistics for a two-tailed test. Any lag data point that lies outside this significance range is deemed an outlier and also removed. For the number of lag data points, this condition has a much stricter rejection criterion than, for example, Chauvenet's criterion. These two steps appear to be a highly robust way to remove erroneous data points. A linear function is then fit to the remaining lag data points and the propagation speed of the wave can be obtained from the gradient, with the error on the gradient calculated using the sample standard deviation.

For the propagation speed maps (Fig. 4), the time–distance diagrams used have varying spatial lengths depending on the position of the pixel under consideration. The standard length is 25 spatial pixels but can be shorter, for example, for pixels near the occulting disk.

Note that the inward propagation speeds suffer from great uncertainty in the lowest part of the corona. Typically this results in unphysically large values for the propagation speed that are well in excess of the outward propagation speed and the estimated Alfvén speed using the PFSS estimates for magnetic field and density measurements from CoMP (Fig. 5a). It can be seen in the average power spectra (Fig. 3) that the amplitude of the inward propagating waves is comparable to the noise level for the higher frequencies. This has the greatest influence in the lower corona where amplitudes of waves are smaller (Fig. 4a–e). The amplitude of both inward and outward waves grow with height above the limb (Fig. 4d) and at a certain point the inward wave amplitude becomes large enough to overcome the noise and provide reliable measurements. In addition, the time–distance diagrams for pixels of the lower corona will typically have less data points spatially, as mentioned, which leads to greater uncertainties in the results. For this reason, the propagation speeds are clipped at 600 km s^−1^ and this predominantly affects the inward wave speed (Fig. 4b).

### Density measurements

CoMP is able to measure both the 10,798 Å and the 10,747 Å Fe XIII emission lines, the ratio of which is known to be sensitive to the electron number density, *n*_e_, in the corona^66^. At 20:29 UT, after the main 10,747 Å sequence was taken, CoMP was set to take scans at both 10,798 Å and 10,747 Å. The scans are used to calculate the central intensity for each wavelength and the intensity ratio is calculated for a number of consecutive frames and averaged. Then, using the CHIANTI database v7.0 (ref. 67), electron density versus intensity ratio curves are calculated for a range of heights above the photosphere taking into account the strong influence of photo-excitation on the formation of the two lines. The curves are then used to calculate the electron number density in the open-field region, which can be converted to coronal mass density using *ρ=μm*_p_*n*_e_, where μ is the mean atomic weight (1.3 for coronal abundances) and *m*_p_ is the proton mass, 1.67 × 10^−27^ kg. The electron density measurements as a function of height (stars) are shown in Fig. 5b and compared with a scaled analytic density profile (dashed line—ref. 7). The measurements only cover a relatively short section of the corona due to weak emission in the corresponding region in 10,798 Å. As the emission decreases in strength with height, the associated errors increase and the number of pixels with significant signal decreases. Both these factors are likely to be responsible for the increased deviation in measured density from the analytic profile with height.

### Estimating quantities from magneto-seismology

In the main text, we discussed the process of estimating the flow speed in the corona via magneto-seismology of the solar atmosphere by exploiting the measured propagation speeds of the waves. This relationship can be obtained from the following equation:


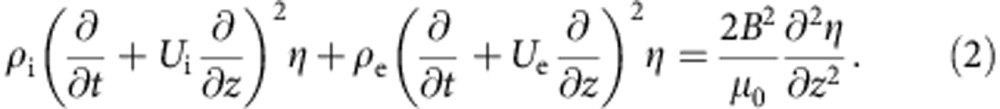


Here, *η* is the perturbation of the displacement, *μ*_0_ is the magnetic permeability of free space, and the subscript i and e correspond to quantities inside and external to the waveguide, respectively. The magnetic field inside and external to the waveguide is assumed equal. This equation describes the kink wave in a thin over-dense waveguide in a low-beta plasma^68^. The condition for the waveguide to be thin is satisfied if the tube radius is much less than the wavelength. This is satisfied for the current observations where *λ*∼100 Mm. Looking for plane wave solutions and under the assumption that *U*_i_*≈U*_e_, it is straightforward to arrive at equation (1), with *c*_ph_ equal to the kink speed. The square of the kink speed is given by *c*_k_^*2*^=*B*^*2*^*/(μ*_0_*<ρ>)*, where *<ρ>*=*(ρ*_i_*+ρ*_e_)/2 is the average density.

Further information about coronal plasma properties can also be obtained via magneto-seismology of the solar atmosphere, if we exploit measurements of the wave amplitude and propagation speed together. In particular, gradients in the density and magnetic field strength can be estimated^69^,^70^. The results can then be compared with estimates from complementary techniques, that is, the magnetic field from PFSS extrapolations; density from CoMP line ratios and the analytic model from ref. 7.

MHD wave theory reveals that the relationship between the measureable quantities and the quantities to be estimated is given by,


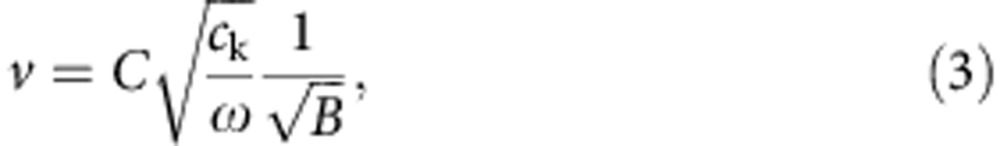


where *v* is the velocity perturbation amplitude of the wave, *ω* is the angular frequency, *C* is a constant and *B* is the magnetic field strength. Due to the presence of the unknown *C*, it is only possible to find the gradients of the density and magnetic field strength. The magnitude of the quantities can only be known if we have initial values for both the quantities. Hence, the gradient of each quantity is normalized and can be scaled for comparison with the magnetic field and density estimates measured by other means.

To exploit equation (3), the propagation speed is averaged horizontally over the boxed region shown in Figs 2 and 4 and plotted as a function of height in Fig. 5a.

In addition to the propagation speed, the Doppler velocity amplitude is also required as a function of height. Each velocity time series in the boxed regions is smoothed using boxcar function of length 3. The root mean square (RMS) value of the removed signal due to smoothing is found to match the estimated RMS of the noise, implying that the smoothing isolates the real velocity signal and suppresses the noise. Taking the RMS value of the smoothed velocity signal and averaging, we obtain the Doppler velocity displayed in Fig. 4e (stars).

Now, the variation in velocity amplitude for Alfvénic waves is expected to be due to changes in density alone and is given by *v*∝*n*_e_^−*1/4*^ (assuming electron and ion number densities are equal and wave damping is negligible). Using this expression, the density gradient inferred from the RMS Doppler velocity amplitude (red diamonds) shows good agreement with the analytic density profile (Fig. 5b). Note that the density gradient determined from the Doppler velocity has been scaled for comparison with the analytic density profile and the density values obtained from the CoMP line ratios.

Using the measured Doppler velocity amplitude and propagation speed, the gradient in magnetic field is obtained using equation (3) and shown in Fig. 5c (red diamonds). We can compare this to the average magnetic field strength in the open-field region estimated from the SDO/Helioseismic and Magnetic Imager magnetograms via the PFSS extrapolation (dashed line). As we are unable to provide an exact boundary for the open-field region, the PFSS magnetic field is examined when averaged over latitudes >80 degrees and >85 degrees, both results incorporate the open magnetic flux region seen in Fig. 1. The results from both latitude boundaries show minor differences, mainly near the solar surface and are practically identical in the corona. It can be seen that there is good agreement between the two measures for the gradients of the magnetic field. Note that, again the magneto-seismological profile has been scaled to allow comparison with the PFSS profile.

The PFSS and magneto-seismology results suggest that the magnetic field strength decreases only by ∼0.5 Gauss from 1.05 to 1.2 
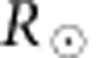
. However, the magnetic field strength in the corona is weak, which means that this corresponds to a substantial decrease of 30–50%.

Finally, to complete the comparison between the different measures, the PFSS magnetic field and analytic density profile are combined to provide an estimate for the averaged Alfvén speed (dashed line—Fig. 5a). As noted in the main text, the agreement between magneto-seismology results and the PFSS and line ratio measurements provides re-assurance that the plasma properties of this open magnetic field region are well constrained.

### Non-thermal widths

The non-thermal widths of the spectral lines are often used as an indicator of Alfvénic wave activity^20^,^21^,^22^,^23^,^24^,^25^, so we also derive them to provide a comparison with the Doppler shift velocities. The non-thermal widths are also calculated from the Fe XIII emission line profile. The peak formation temperature of Fe XIII is ∼1.6 MK, so it is expected that the line profile has a thermal width *σ*_T_*=√(2k*_b_*T*_e_*/m*_ion_)=21 km s^−1^, where *k*_b_ is the Boltzmann constant, *T*_e_ is the electron temperature (assumed equal to the ion formation temperature) and *m*_ion_ is the mass of the ion. In addition, the line will be broadened by an instrumental width that has a value of 21 km s^−1^. The thermal and instrumental widths can be removed from the measured Doppler width to provide the non-thermal width. As with Doppler velocity, the non-thermal width is averaged horizontally over the boxed region and plotted as a function of height in Fig. 4e. The gradients in the amplitude of both the Doppler velocities and non-thermal widths are found to be approximately equal, demonstrating that both are measures of Alfvénic wave activity in the corona.

### Wave energy flux

SDO/AIA is used to measure the swaying motions of the magnetic field, which can be interpreted as MHD kink waves. The magnetic field is outlined by density enhancements in the corona seen in the open-field region (Fig. 1d). The measurements are performed at a height of ∼1.01 
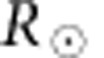
 and follow the methods in ref. 31. The results are shown as histograms in Fig. 6. A median value for the sample population of each quantity, that is, displacement amplitude, period and velocity amplitude, is calculated. The distributions for each of these quantities show approximately log-normal behaviour; hence, the mean value of the log-normal distribution is calculated to give the average values. These values are given in Table 2.

To estimate the wave energy flux, we require an estimate for the mass density in the corona. This is determined from extrapolating from the CoMP electron density measurements back to 1.01 
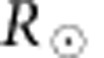
 using the scaled analytic function^7^. The value of the mass density used is then *ρ*=9.5 × 10^−13^ kg m^−3^. In addition, the phase speed at 1.01 
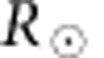
 is taken from the estimate of average Alfvén speed in ref. 30.

The wave energy flux from the non-thermal widths uses the density and value for non-thermal widths at a height of 1.06 
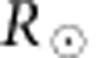
.

## Additional information

**How to cite this article:** Morton, R. J. *et al*. Investigating Alfvénic wave propagation in coronal open-field regions. *Nat. Commun*. 6:7813 doi: 10.1038/ncomms8813 (2015).

## Figures and Tables

**Table 1:** Properties of the 10^a^ f^b^ fit to the outward (Out) and inward (In) power spectra shown in Fig. 3, for the box (M1) and track (M2) methods.

	Frequency range (mHz)	*a*	*b*	*χ*_ν_^*2*^
Out M1	0.2–2	−5.0±0.1	−0.92±0.03	4.3
	4–10	−4.8±0.1	−1.04±0.05	3.8
In M1	0.2–2	−5.3±0.1	−0.98±0.03	3.8
Out M2	0.2–2	−4.4±0.1	−0.7±0.04	0.9
	4–10	−5.0±0.1	−1.1±0.05	1.4
In M2	0.2–2	−5.1±0.1	−0.89±0.04	2.7

**Table 2:** Measured wave properties from SDO/AIA data.

	Median	Mean from log-normal
Displacement (km)	470	590±5
Period (s)	268	414±5
Velocity (km s^−1^)	11.2	14.7±0.2
